# Bridging Gaps in Disaster Nursing Education: A Qualitative Study of Barriers and Opportunities in Indonesia

**DOI:** 10.1002/nop2.70647

**Published:** 2026-06-16

**Authors:** Etika Emaliyawati, Kusman Ibrahim, Yanny Trisyani, Praneed Songwathana, Restuning Widiasih, Didi Setiadi, Firman Sugiharto

**Affiliations:** ^1^ Department of Critical Care Nursing and Emergency Nursing, Faculty of Nursing Universitas Padjadjaran Bandung West Java Indonesia; ^2^ Department of Medical‐Surgical Nursing, Faculty of Nursing Universitas Padjadjaran Bandung West Java Indonesia; ^3^ Faculty of Nursing Prince of Songkla University Songkhla Thailand; ^4^ Department of Maternity Nursing, Faculty of Nursing Universitas Padjadjaran Bandung West Java Indonesia; ^5^ Research Center for Climate and Atmosphere National Research and Innovation Agency Jakarta Indonesia; ^6^ Faculty of Nursing Universitas Padjadjaran Bandung West Java Indonesia

**Keywords:** barriers, disaster nursing, nursing education, opportunities

## Abstract

**Aims:**

This study aimed to explore the barriers and potentials in disaster nursing learning in undergraduate nursing study programmes in Indonesia.

**Design:**

A descriptive qualitative study.

**Method:**

The study was carried out from March 2019 to March 2020. A purposive sampling technique was used to recruit 54 participants, consisting of lecturers, students, disaster volunteers and members of emergency and disaster nursing associations. Data were collected through focus group discussions and analysed using thematic analysis to identify the main themes.

**Results:**

There are three main barriers in disaster nursing learning, namely: (1) curriculum and competency standards are not uniform, (2) limited facilities and infrastructure and (3) limited understanding and readiness of lecturers. On the other hand, several strategic opportunities were also found, such as the (1) high frequency of disasters as a source of real learning, (2) synergy between educational institutions and professional associations and (3) student involvement in disaster activities through student activity units and independent campus programmes.

**Conclusion:**

The results of this study emphasize the need for developing a standardized disaster nursing curriculum, increasing the capacity of lecturers and integrating experience‐based learning methods to improve the readiness of nursing students to deal with disaster situations effectively.

## 1 Introduction

Disaster nursing education is important in preparing nurses to deal with disaster emergencies (Eid‐Heberle and Burt 2023). In the context of Indonesia, which is located in a disaster‐prone area, the role of nurses is crucial in providing health services to affected communities (Martono et al. 2019). Although nurses are on the front lines of disaster response, many face significant challenges related to disaster management preparedness and training. According to the International Council of Nurses, nurses must have nine basic disaster nursing competencies (Internasional Council of Nursing 2009). Core competencies in disaster nursing encompass essential skills and knowledge across preparedness, communication, incident management, safety, assessment, intervention, recovery and ethical–legal practice to ensure effective nursing response in disaster situations (Internasional Council of Nursing 2009). This competency is undoubtedly important for nurses since they are in the education process.

The frequency and intensity of disasters in Indonesia continue to increase; data shows that in 2024 there will be 1270 disaster incidents (National Board for Disaster Management n.d.). In addition, Indonesia has experienced the COVID‐19 pandemic from 2020 until now, which continues to increase the number of infected patients and even deaths (Ministry of Health of the Republic of Indonesia n.d.). This increase in incidents requires better preparedness of health workers, including nurses, to respond effectively to emergencies.

Although the importance of disaster nursing education has been recognized, nursing education programmes in Indonesia still lack a comprehensive curriculum on disaster management. This is evidenced by several studies conducted in Indonesia showing that nurses' preparedness for disasters is still relatively low (Ihsan et al. 2022; Holifatus Suaida et al. 2024; Oktarina et al. 2024) Previous studies have reported that nurses are underprepared for disasters and do not understand their role in both the disaster preparedness phase and post‐disaster situations (Martono et al. 2019). Previous reviews also concluded that most nurses were unprepared to face disasters, ranging from 46.5% to 97.5% (Ihsan et al. 2022). Nurses in some developing countries are poorly prepared to deal with disasters.

The low level of disaster preparedness among nurses highlights the need for holistic disaster education implementation for nursing students (Eid‐Heberle and Burt 2023; Labrague and Hammad 2024; Achora and Kamanyire 2016). This education aims to prepare them to face diverse disaster challenges more effectively (Loke et al. 2021). However, many nursing programmes in Indonesia do not have a standard curriculum for disaster nursing, leading to educational inconsistencies across institutions (Ituma et al. 2022; Mckenzie 2019). This is a serious concern considering that Indonesia is a disaster‐prone country, with incidents such as earthquakes, floods and forest fires increasing yearly.

The lack of resources and materials specifically designed for disaster nursing education hampers educators' ability to provide comprehensive training (Mckenzie 2019). Then, the implementation of the disaster nursing learning model in Indonesia is still not optimal and understood due to various reasons, such as limited knowledge and readiness of lecturers, facilities and tools that do not support the disaster nursing learning process, so the achievement of disaster nursing learning varies on each campus (Ljunggren and Rosengren 2019). Therefore, further development in disaster management education is needed to improve the preparedness of nurses and nursing students in facing global challenges related to disasters (Loke et al. 2021).

In developing nursing education, it is necessary to evaluate the barriers, challenges, opportunities and needs in disaster nursing learning as an important initial step in this development process (Ituma et al. 2022; Songwathana and Timalsina 2021; Al Thobaity 2024). Through this qualitative study, the authors aim to explore various aspects related to disaster nursing education in undergraduate nursing programmes, including both the barriers that hinder learning effectiveness and the opportunities that can support the improvement of disaster management training. By understanding these dynamics in a balanced manner, educational institutions can develop more targeted and innovative strategies to strengthen disaster preparedness among future nurses, thereby enhancing individual competencies and contributing to a more effective public health response during crises.

## 2 Material and Methods

### 2.1 Study Design

This study employed a qualitative descriptive approach and was conducted between March 2019 and March 2020.

### 2.2 Participants

Participants consisted of disaster volunteers from 30 provinces in Indonesia, members of the Indonesian Emergency and Disaster Nurses Association and representatives from the College of Nursing under the auspices of the Association of Indonesian Nursing Education Institutions. This study involved 54 participants, including 13 lecturers, 16 nursing students, 19 disaster volunteers and 6 policymakers. Participants were selected using purposive sampling to ensure the inclusion of individuals with relevant knowledge, experience and direct involvement in disaster nursing education and practice. The inclusion and exclusion criteria were defined to capture diverse perspectives while maintaining data quality and relevance.

For lecturer participants, the inclusion criteria were: (1) permanent lecturers at accredited nursing higher education institutions in Indonesia and (2) actively teaching disaster nursing courses and willing to participate. Lecturers with < 1 year of teaching experience or from non‐accredited institutions were excluded to ensure adequate academic experience and institutional quality. For student participants, inclusion criteria included: (1) students who had completed disaster nursing courses across different learning periods (pre‐pandemic, during fully online learning and hybrid transition) and (2) willingness to participate. Students who had not completed prerequisite courses were excluded to ensure sufficient foundational knowledge.

For disaster volunteer participants, inclusion criteria were: (1) registered nurses working in healthcare facilities, (2) having at least 1 year of professional experience, (3) direct involvement in disaster response in Indonesia and (4) active participation in emergency and disaster nursing associations. These criteria were applied to ensure participants had practical, field‐based experience. Volunteers aged under 21 years or unwilling to participate were excluded. For policymaker participants, inclusion criteria included: (1) central‐level administrators of the Indonesian Emergency and Disaster Nurses Association, (2) prior experience in disaster response settings and (3) willingness to participate. Policymakers with < 2 years of leadership experience were excluded to ensure adequate policy‐level insight and decision‐making experience.

### 2.3 Data Collection

Data was collected through the focus group discussion (FGD) method guided by the principal researcher (E.E.), an experienced moderator who has taught for over 15 years. The primary researcher is an experienced nursing educator with a background in disaster nursing. FGD was used to explore participants' information, opinions and views regarding problems, barriers, opportunities, challenges, needs, expectations and implementation of learning in the context of disaster nursing. Participants were recruited based on predetermined inclusion criteria and consisted of 13 lecturers, 16 students, 19 disaster volunteers and 6 policymakers.

To minimize potential researcher bias, several strategies were employed. The researcher practiced reflexivity by maintaining awareness of personal assumptions and prior experiences in disaster nursing education throughout the data collection process. An interview guide with open‐ended questions was used to ensure consistency across all FGDs and to reduce leading questions. In addition, discussions were conducted in a neutral and non‐judgmental manner to encourage participants to freely express their perspectives. Field notes were taken during and after each session to capture contextual insights and support data interpretation.

After obtaining consent from the participants and agreeing on the time and place of implementation, the researcher formed discussion groups of 6 to 10 people. Due to the COVID‐19 pandemic, FGDs for lecturers and students were conducted online via Zoom, while FGDs for volunteers and policymakers were conducted in person. Before the discussion began, the researcher explained the study's purpose and procedures and obtained informed consent from all participants by signing a form or filling out a Google Form. The researcher also explained the FGD rules, where each participant was given a number and asked to raise their hand before giving their opinion. During the discussion, the researcher used an open‐ended question guide to ensure the data collected was relevant and in‐depth. The discussion ended after all questions in the guide were answered and deemed sufficient to meet the research needs.

Data saturation was determined through an iterative and concurrent process of data collection and analysis. After each discussion session, the research team conducted preliminary coding and compared emerging categories across participants. Saturation was considered achieved when no new codes, themes or perspectives emerged in at least two consecutive discussions, and when the existing categories were well‐developed, consistent and sufficiently rich to explain the phenomenon under study. This redundancy of information indicated that further data collection would yield minimal additional insights. Through this approach, a comprehensive understanding of the dynamics of disaster nursing learning from multiple perspectives was ensured.

### 2.4 Data Analysis

The data was analyzed using thematic analysis. Thematic analysis is used for descriptive qualitative research that is appropriate when researchers want to understand the perspectives, experiences or meanings expressed by participants (Naeem et al. 2023). Thematic analysis is a method for identifying, analyzing and reporting patterns or themes that emerge from data (Braun and Clarke 2006, 2013).

Qualitative data analysis in this study followed the thematic analysis steps outlined by Guest et al. (2012). The process begins with transcription, which converts the oral data from the FGD recordings into writing, which includes all the words spoken by the participants, including pauses, laughter and other non‐verbal expressions. Next, relevant data is organized and grouped into a format that facilitates analysis. After the transcription is complete, the researcher conducts familiarization by reading the data thoroughly to understand the context and identify patterns and concepts that emerge from the participants' expressions. At this stage, the researcher also makes initial notes about the thoughts expressed.

The next stage is data coding, where researchers mark text sections relevant to the research topic and identify words, phrases or sentences that fit the questions asked during the FGD. These codes then become initial categories. After that, researchers identify themes by grouping similar codes and re‐examining the data repeatedly to ensure consistency. The theme review process is carried out to ensure that the identified themes are relevant and consistent, and content analysis is conducted to identify patterns or meanings. Once the themes are established, researchers name and define the themes in detail to reflect the essence of the data contained while paying attention to the context and interpretation of the results related to the research questions. In the final stage, researchers compile a report presenting the main findings and direct quotes from FGD participants to support the research results. The report also includes a discussion of the findings in the context of existing literature.

### 2.5 Rigour and Trustworthiness

This study was reported in accordance with the Consolidated Criteria for Reporting Qualitative Research (COREQ) checklist to ensure transparency and rigour in qualitative reporting. In addition, credibility, dependability, confirmability and transferability are used to ensure rigour and trustworthiness (Lincoln and Guba 1985). Credibility is achieved through member checking, where themes are derived from participant data and presented to participants to obtain feedback. Transferability is achieved by applying phenomenological methods to apply the results to other situations with similar contexts. Dependability is achieved by applying descriptive qualitative methods and analyzing data using thematic analysis to obtain a complete description of the phenomenon being studied. Finally, confirmability is achieved through the researcher's reflection on the findings with related scientific articles, consultation with experts or specialists in their fields, and presenting the findings at research seminars (Lincoln and Guba 1985). In the end, there was a standard view and opinion regarding the findings from expert researchers.

### 2.6 Ethical Considerations

This research was conducted based on the principles of the Declaration of Helsinki (World Medical Association 2013).

## 3 Results

### 3.1 Participants Characteristics

Participants in this study consisted of four categories, namely nursing lecturers in Indonesia, nursing students, disaster volunteers and administrators of emergency and disaster nursing organizations (Table 1). The characteristics of participants across all groups demonstrate several dominant patterns. Among lecturers (*n* = 13), the majority were male (69.23%) and affiliated with public universities (69.23%). The most prevalent age group was 41–50 years (38.46%), and most had 11–20 years of teaching experience (38.46%).

**TABLE 1:** Research participants (*n* = 54).

Participant categories	Frequency (f)	Percentage (%)
Lecturers (*n* = 13)		
Gender		
Male	9	69.23
Female	4	30.77
Institutions		
Public universities	9	69.23
Private universities	4	30.77
Age (year)		
31–40	4	30.77
41–50	5	38.46
51–60	4	30.77
Experience as a lecturer (year)		
1–5	3	23.08
6–10	2	15.38
11–20	5	38.46
> 20	3	23.08
Nursing students (*n* = 16)		
Gender		
Male	1	6.25
Female	15	93.75
Disaster volunteers (*n* = 19)		
Gender		
Male	19	100
Female	0	0
Institutions		
Hospitals	11	57.89
Primary healthcare	1	5.26
University	7	36.85
Age (year)		
21–40	9	47.37
41–50	8	42.11
51–60	2	10.52
Volunteer experience		
1–5×	15	78.95
6–10×	3	15.79
> 11×	1	5.26
Association central executive (*n* = 6)		
Gender		
Male	5	83.33
Female	1	16.67
Institutions		
Services/Hospital	3	50
Education	2	33.33
Independent institution	1	16.67
Age (year)		
40–45	4	66.67
> 45	2	33.33

Among nursing students (*n* = 16), the sample was overwhelmingly female (93.75%). In the disaster volunteer group (*n* = 19), all participants were male (100%), with the majority affiliated with hospitals (57.89%). The most represented age group among volunteers was 21–40 years (47.37%), and most had 1–5 times volunteer experience (78.95%). For participants from the association central executive (*n* = 6), the majority were male (83.33%) and primarily worked in service or hospital settings (50%). Most were aged between 40 and 45 years (66.67%), representing the largest proportion within this group.

### 3.2 Barriers to Disaster Nursing Education

**FIGURE 1:**
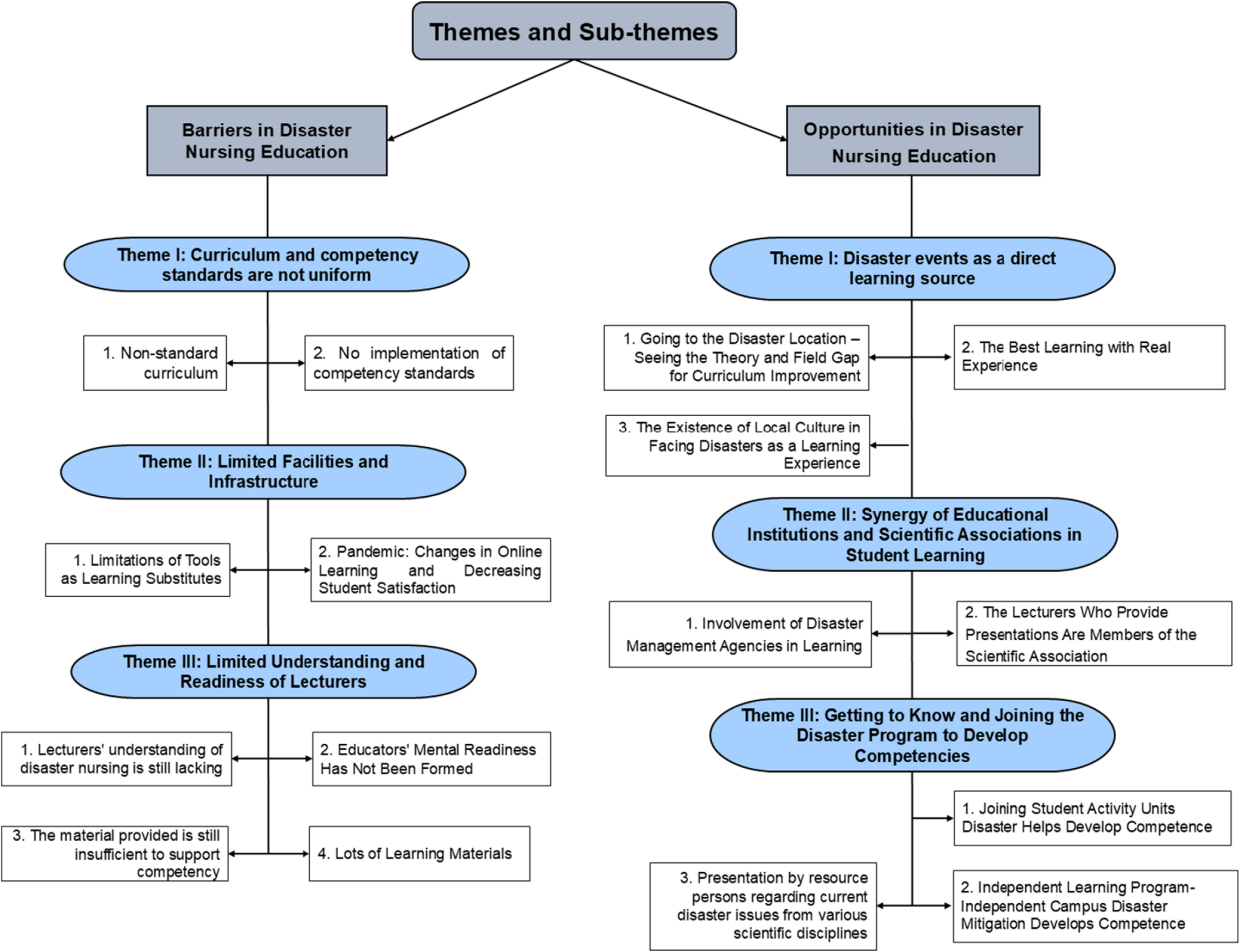
Barriers and opportunities to disaster nursing learning.

This study identifies various barriers faced in disaster nursing education in Indonesia based on the experiences and views of informants, including nursing lecturers, students, disaster volunteers and administrators of professional organizations. The thematic analysis results indicate several main issues that are barriers to the optimal implementation of disaster nursing education. Three main themes that emerged in this study are: (1) curriculum and competency standards are not uniform, (2) limited facilities and infrastructure and (3) limited understanding and readiness of lecturers (See Figure 1).

#### 3.2.1 Theme I: Curriculum and Competency Standards Are Not Uniform

This theme illustrates that there is no uniform curriculum and competency standards for disaster nursing across all educational institutions in Indonesia. The results of the FGD illustrate that the learning approach and understanding of disaster nursing are still very diverse, even tending to be partial. The results of the FGD with nursing lecturers in various regions of Indonesia showed that most institutions do not yet have a comprehensive disaster nursing curriculum. One lecturer said:

The curriculum should ideally cover all phases, not just focus on the emergency response phase…. (FGD.1.R.D.12)

This shows that most learning is still focused on the emergency response phase without emphasizing the importance of knowledge and skills in pre‐ and post‐disaster phases. In addition, limitations in real practical experience are also significant barriers. As expressed by a lecturer:

‘We have never conducted simulations or gone directly to disaster sites…’ (FGD.1.R.D.5), and ‘We have not implemented tabletop exercises yet… for practicum… it still has not been realized…’ (FGD.1.R.D.11)

These statements reflect the gap between planning and actual field practice and the minimal application of learning methods, such as tabletop exercises essential in mastering strategic disaster management. The lack of competency standards was also highlighted in FGDs with disaster volunteers. One participant stated:

If we look at it, the curriculum for the competencies nurses need in disaster management has not yet been standardized. (FGD.2.R.RB.4)

In line with that, another participant stated:

‘Many nurses still lack disaster management competencies—whether in the pre‐, during‐, or post‐disaster phases.’ (FGD.2.RB.7), and ‘Campuses often teach local disaster scenarios, like earthquakes or fires, not just tsunamis, but still not covering the entire disaster management system’. (FGD.2.R.RB.6)

This shows that the material taught on several campuses is still limited to types of local disasters without a comprehensive discussion of the disaster management system. Similar findings also emerged in the FGD with the central management of the emergency and disaster nursing professional organization. One participant stated:

There is no standard curriculum for higher education… currently, every campus has a different one. (FGD.3.R.PPH.2)

Another added: Students' competencies are lacking… we teach in normal conditions, with available facilities. However, disaster situations are not like that. We need to train for alternatives… Another aspect lacking is resilience, the ability to endure and survive. (FGD.3.R.PPH.5)

The gap between everyday learning contexts and real disaster situations is one of the barriers to building nurse resilience. Furthermore, other participants highlighted the variation in understanding between campuses:

‘From what I have seen, campus perspectives vary widely. Many still view disaster nursing as just emergency response’. (FGD.3.R.PPH.1), and ‘The difficulty is… disaster nursing is not yet structured’. (FGD.3.R.PPH.1)

This explanation shows that most institutions still equate disaster nursing with emergency nursing, thus blurring the focus of learning and the competencies that should be developed.

The most substantial criticism came from a professional board member who stated directly:

So far, we still do not have a competency standard, sir. (FGD.3.R.PPH.4)

This reflects the weakness of structural support and national policies in developing disaster nursing education, which results in graduates who are unprepared to engage in real disaster situations.

#### 3.2.2 Theme II: Limited Facilities and Infrastructure

This theme highlights the limitations of facilities and infrastructure that are the main barriers to disaster nursing learning in educational institutions. The availability of supporting tools and facilities, such as simulation equipment, disaster laboratories, and field practice facilities, is still inadequate. This condition is a serious barrier to achieving the competencies targeted in the curriculum, especially those related to direct practice skills, simulations and decision‐making in disaster situations.

##### 3.2.2.1 Limitations of Tools as Learning Substitutes

In implementing disaster nursing learning, participants from among lecturers and administrators of professional organizations said that most educational institutions do not yet have adequate simulation tools, including important devices such as tabletop exercises. The unavailability of these tools makes disaster management training difficult to implement optimally, so students do not have sufficient experience in understanding the dynamics of real disaster situations. One participant said:

…Even table top simulation tools are not available… (FGD.1.R.D.12)

Another lecturer added:

We have not conducted simulations yet… our disaster tools are inadequate. (FGD.1.R.D.3)

This statement was reinforced by participants from the central management of the emergency and disaster nursing organization, who stated:

Materials or tools as substitutes are still very lacking… (FGD.3.R.PPH.5)

These quotes show that the limitations of teaching tools and materials are a significant barriers in the disaster nursing learning process. This creates a gap between theory and practice that hinders the achievement of learning outcomes.

##### 3.2.2.2 Pandemic: Changes in Online Learning and Declining Student Satisfaction

In addition to the limitations of physical facilities, the COVID‐19 pandemic has also worsened the learning situation by forcing institutions to switch entirely to online methods. In disaster nursing, which emphasizes direct experiential learning, students have experienced a significant decline in the quality and effectiveness of learning. A student said,

…there were no offline activities, and that is what is unfortunate. (FGD.4.R.M.7)

Another participant added,

…because of the pandemic too, ma'am, so it could not be forced. (FGD.4.R.M.4)

This condition makes students lose the opportunity to feel a disaster's real atmosphere, crucial in forming intuition, situational sensitivity and quick response. This was clearly expressed by one of the students, who stated:

The lack might be, maybe we haven't experienced the actual practice… we have not felt the atmosphere, what it is really like in a disaster situation… so we have not experienced it, and we are still curious… want to feel it, really want something like that… (FGD.4.R.M.6)

The lack of practical experience makes students feel less confident in their abilities, as expressed:

… what is concerning is the practice, ma'am. So, the worry is about our competency during, uh… during disaster management practice. (FGD.4.R.M.6)

This statement emphasizes that online methods have not been able to replace direct experience‐based learning. Without real practical experience, students lose not only technical skills but also the confidence that is important in making decisions in the disaster field.

#### 3.2.3 Theme III: Limited Understanding and Readiness of Lecturers

This theme reveals the limitations of lecturers' understanding and readiness to teach disaster nursing comprehensively. Four sub‐themes support this finding, namely: (1) lecturers' understanding of disaster nursing is still lacking, (2) educators' mental readiness has not been formed, (3) the material provided still does not support competency, and (4) there is a lot of learning material.

##### 3.2.3.1 Lecturers' Understanding of Disaster Nursing Is Still Lacking

The limited understanding of lecturers is the main challenge in implementing disaster nursing learning. A volunteer lecturer said:

…one issue is about human resources, ma'am. For human resources, not all lecturers may fully understand disaster nursing yet. (FGD.1.R.D.3)

This statement shows that not all lecturers comprehensively understand disaster nursing. This can also be seen from the lack of understanding of lecturers towards learning methods such as tabletop exercises, as stated by the following participant:

…about the barriers earlier, ma'am. We still do not understand the tabletop method for, uh… disaster. (FGD.1.R.D.6)

Disaster volunteers also conveyed gaps in understanding:

…regarding the boundaries of when RHA (Rapid Health Assessment) is conducted, up to which day lecturers need to understand that first. (FGD.2.R.RB.3)

Lecturers' lack of knowledge about RHA procedures indicates a need for further training for nursing educators on emergency protocols and practices.

A representative from the central association board expressed another opinion:

…and then the understanding at many campuses I have seen… varies a lot. They think disaster is just about emergency response… many campuses approach disaster nursing as just emergency care. (FGD.3.R.PPH.1)

This statement reflects a misconception among educators in academic institutions who view disaster nursing as limited to emergencies. This highlights a limited perspective, whereas disaster nursing should encompass various stages of disaster management, including mitigation, preparedness, response and recovery.

##### 3.2.3.2 Educators' Mental Readiness Has Not Been Formed

In addition to the lack of understanding, lecturers' mental readiness as disaster nursing educators has not been fully formed. Disaster volunteers conveyed this:

…our mental readiness as educators has not yet been formed… it should be there already since there is a curriculum. (FGD.2.R.RB.16)

Although a curriculum exists, there remains a gap between its content and the mental readiness of educators to teach it. This shows that curriculum development needs to be accompanied by training and efforts to strengthen educators' readiness to fulfil their roles optimally.

##### 3.2.3.3 The Material Provided Is Still Insufficient to Support Competency

Participants also highlighted that disaster nursing learning materials provided in educational institutions do not fully support students' practical competencies. As expressed by the following disaster volunteer:

…I noticed in the field that the equipment is not well prepared… when responding to a disaster, physical and mental readiness and the equipment must all be ready. This often happens in the field and must be addressed through education—that is education's job… (FGD.2.R.RB.10)

…they forget to take part in management support so that the management becomes chaotic and no one coordinates… the ability in management support is still lacking, and nurses focus more on medical support. (FGD.2.R.RB.8)

…another area that is still quite lacking is resilience, endurance, survival skills. (FGD.3.R.PPH.5)

These statements highlight the importance of learning that prepares students clinically, physically, mentally and in terms of management and resilience skills. Many students are not equipped with adequate survival and leadership skills to face real‐life challenges in the field.

##### 3.2.3.4 Lots of Learning Materials

Students expressed that the material burden in disaster nursing learning felt very heavy, especially during the pandemic where learning was carried out online:

The material is quite a lot, yeah. So, uh, for midterms and finals, when the materials are combined, it can be a lot… even though some of the material is easy to understand, some of it is hard to grasp. (FGD.4.R.M.4)

The large amount of material that must be mastered in a short time makes students feel overwhelmed, especially without the support of direct interaction with lecturers. This impacts their level of satisfaction with their learning and their confidence in mastering disaster nursing competencies.

### 3.3 Opportunities in Disaster Nursing Education

This study also identified various opportunities for developing disaster nursing education in Indonesia based on the results ofFGDs with lecturers, disaster volunteers and administrators of professional organizations. Thematic analysis revealed that Indonesia's geographical and cultural context, which is prone to disasters, is an advantage in forming learning based on real experiences. Three main themes that represent opportunities in disaster nursing education are: (1) Disaster events as a source of direct learning, (2) Synergy between educational institutions and scientific associations in student learning and (3) Knowing and joining disaster programmes.

#### 3.3.1 Theme I: Disaster Events as a Direct Learning Source

This theme emerged from understanding the high frequency of disaster events in Indonesia. Indonesia's diverse and frequent disaster events are considered an important opportunity for disaster nursing learning. Participants conveyed:

The disaster in our area, the Sinabung eruption, can be used as a learning tool… (FGD.1.R.D.7)

…during the Lombok earthquake… our disaster nursing department was also involved. We saw many shortcomings… (FGD.1.R.D.8)

Direct experience from being involved in disaster management also opened the eyes of educators to the real deficiencies and challenges in the field. Disaster volunteers expressed similar things:

With the frequent disasters, we involve students in disaster situations… (FGD.2.R.RB.17)

From the central management of the emergency and disaster professional association, emphasis emerged on the importance of solidarity and community empowerment as unique social learning:

…the frequent disasters here have become learning experiences—empowering groups to help one another, this closeness is Indonesia's strength; other countries do not have this… (FGD.3.R.PPH.2)

Disasters often serve as valuable learning experiences for lecturers and volunteers, enhancing their capacity while involving students directly in crises. This active participation opens opportunities for evaluating and improving the curriculum to align with real‐world needs. Students also benefit by gaining hands‐on experience, strengthening their competence and preparedness.

##### 3.3.1.1 Going to the Disaster Location—Seeing the Theory and Field Gap for Curriculum Improvement

Direct involvement of educators and students in disaster zones highlights the gap between theory and practice, prompting reflection on making learning more contextual and applicable. A lecturer shared:

…our department also went down to the field. We saw many shortcomings and gaps between theory and practice. So we started to redesign the disaster nursing curriculum together… (FGD.1.R.D.8)

Volunteers also emphasized the importance of diverse disaster types in course content:

…in campuses, local disaster studies often include not only tsunamis, but also earthquakes, fires, and other hazards. (FGD.2.R.RB.6)

Students actively involved in disaster management with campus organizations also gained valuable experience:

…involving students in disaster conditions through student organizations has many benefits… (FGD.2.R.RB.17)

This experience bridges theory and practice, enriching students' understanding and helping educators develop a curriculum that is more contextual and adaptive to various types of disasters.

##### 3.3.1.2 The Best Learning With Real Experience

Participants from the central management of the Emergency and Disaster Nurses Association emphasized that the most effective learning occurs through direct experience in the field:

…with so many disasters, students can learn through real experiences around them… but this is still underutilized. (FGD.3.R.PPH.5)

Experiencing disaster situations firsthand sharpens students' skills in decision‐making, teamwork and stress response. While real‐world learning has enormous potential, it has not yet been fully integrated into formal education—making it essential to include it in order to prepare disaster‐ready healthcare workers.

##### 3.3.1.3 The Existence of Local Culture in Facing Disasters as a Learning Experience

As a disaster‐prone country, Indonesia has communities that adapt and develop survival methods rooted in local culture. This was reflected in statements from association leaders:

Our frequent disasters provide lessons, and we share experiences about how to overcome disasters. In Simeulue Island, there is a cultural response called “smong.” When there is an earthquake, people shout “smog” and run to the hills. That does not exist elsewhere—it's a powerful local characteristic… (FGD.3.R.PPH.2)

This local wisdom not only saves lives but also reflects community identity. Integrating it into disaster nursing education offers students a contextual understanding that goes beyond theory and technology.

#### 3.3.2 Theme II: Synergy of Educational Institutions and Scientific Associations in Student Learning

Collaboration between academic institutions and professional associations is vital as disaster nursing evolves with global and regional dynamics. This ensures that teaching materials remain relevant and practice‐oriented. Volunteers highlighted this synergy:

… there is a synergy between educational institutions and emergency and disaster nursing associations… having one command structure helps associations directly coordinate with institutions to involve students in fieldwork. (FGD.2.R.RB.17)

This partnership improves the quality and effectiveness of learning by connecting students to real‐world practice.

##### 3.3.2.1 Involvement of Disaster Management Agencies in Learning

Agencies such as BPBD (Regional Disaster Management Agency), Basarnas (Search and Rescue Agency) and other organizations play a key role. Nursing lecturers said:

… we have coordinated with BPBD to deliver disaster content… (FGD.1.R.D.2)

…BPBD and Basarnas support us because we have MOUs with them… (FGD.1.R.D.8)

…we also work with ambulance services to provide theory and practice sessions… (FGD.1.R.D.3)

Students acknowledged how this collaboration broadened their perspective:

…I realized that nursing is not just linked to one stakeholder—there are many involved. (FGD.4.R.M9)

…we had guest lectures from outside—very eye‐opening! It showed how broad and essential nursing is. (FGD.4.R.M6)

…the guest speakers were amazing… they gave us new insights. (FGD.4.R.M7)

This shows that learning is enriched by formal teaching and real‐world contributions from field experts.

##### 3.3.2.2 The Lecturers Who Provide Presentations Are Members of the Scientific Association

Lecturers who are also members of the Indonesian Emergency and Disaster Nurses Association bring added value to teaching:

…for nursing materials, we involve colleagues from the nursing association, not BPBD, to deliver content. (FGD.1.R.D.6)

Being part of such associations grants access to updated content, ongoing training and broad professional networks, which improve teaching quality and help ensure that students learn the latest in disaster nursing.

#### 3.3.3 Theme III: Getting to Know and Joining the Disaster Programme to Develop Competencies

This theme highlights how student involvement in disaster‐related programmes, through Student Activity Units (SAU), the Independent Learning–Independent Campus (ILIC) programme and exposure to interdisciplinary speakers, contributes to competence development.

##### 3.3.3.1 Joining SAU Disaster Helps Develop Competence

SAUs focused on disaster preparedness play a significant role. One lecturer stated:

…our students gain extra skills through campus activities like the Siaga Ners club, which supports disaster response. (FGD.1.R.D.4)

Other lecturers also reinforced this statement:

…Campus X has a CFHC program to boost student abilities before entering the field. Disaster response is not easy without prior learning or simulations. (FGD.1.R.D.1)

Volunteers echoed this:

…involving students in disaster situations through SAUs and associations provides real benefits—they are eager and curious. (FGD.2.R.RB.17)

…emergency and disaster nursing associations always invite student participation—Siaga Ners is one such SAU empowered by the professional association. (FGD.2.R.RB.12)

Students confirmed this impact:

…those who join SAUs can better visualize and understand disaster practice. (FGD.4.R.M6)

…I absorbed disaster‐related materials better in SAU than in class… (FGD.4.R.M7)

This shows that SAU involvement is a highly effective learning strategy, offering practical experience alongside theory.

##### 3.3.3.2 Independent Learning Programme‐Independent Campus Disaster Mitigation Develops Competence

ILIC is a government programme that allows students to study beyond their primary curriculum. A student shared:

…even in my seventh semester, I joined the Merdeka Campus program on disaster mitigation. It was very exciting! (FGD.4.R.M4)

This programme allows students to explore disaster mitigation holistically through academic and field experience, strengthening their planning and preparedness skills.

##### 3.3.3.3 Presentation by Resource Persons Regarding Current Disaster Issues From Various Scientific Disciplines

In addition to SAU and ILIC, students benefit from exposure to expert speakers from various disciplines—both academics and practitioners. Students shared:

…we had project‐based learning and external lectures—it really opened my eyes to how broad and essential nursing is. (FGD.4.R.M6)

…the guest speakers were amazing and provided lots of new information. (FGD.4.R.M7)

Such exposure deepens students' understanding of disaster management from medical, psychological, social and managerial angles, helping them grasp the full scope of disaster preparedness and response.

## 4 Discussion

Disaster nursing education is crucial in preparing competent and resilient health workers to deal with emergencies and disasters (Loke et al. 2021). Considering that Indonesia is a disaster‐prone country, strengthening the learning aspect in this field is very important (Emaliyawati, Ibrahim, Trisyani, et al. 2025). However, until now, disaster nursing education's curriculum development and implementation still face various challenges. Therefore, it is important to understand the barriers and opportunities from various stakeholder perspectives to formulate more effective and responsive learning strategies.

This study explores the barriers and potentials of disaster nursing education in Indonesia based on the perspectives of students, educators and disaster volunteers. The results of this study reveal various barriers and opportunities in disaster nursing learning in Indonesia, which reflect the complexity and urgency of strengthening the curriculum in this field.

### 4.1 Barriers to Disaster Nursing Education

This study found that the main challenges in disaster nursing education in Indonesia include three main things: the absence of standardization of curriculum and competencies, limited learning facilities and infrastructure, and the lack of understanding and readiness of lecturers in delivering disaster nursing materials. From the lecturer's perspective, one of the main barriers is the absence of standardized learning methods and processes. This is reflected in the limited teaching aids, minimal resources and lecturers' limited understanding of disaster nursing. The same barriers occur in Saudi Arabia and Turkey, where simulations and exercises are less than optimal, educational programmes are inadequate, a lack of formal education, difficulty following instructions and difficulty accessing the latest educational resources related to disaster nursing (Kalanlar 2019; Brinjee, Al Thobaity, Al Alahmari, et al. 2021; Brinjee, Al Thobaity, Almalki, et al. 2021).

Several lecturers and disaster nurse associations stated that the current curriculum does not cover all stages of disaster management. These findings are consistent with previous international studies indicating that disaster nursing education remains fragmented and insufficiently integrated into nursing curricula (Brinjee, Al Thobaity, Almalki, et al. 2021; Rostami et al. 2023; Zhang et al. 2024). Limited practical training and lack of standardized competency frameworks have also been widely reported as major barriers affecting students' preparedness (Kaya and Erdoğan 2024; Farokhzadian et al. 2024). The ideal nursing curriculum balances theoretical knowledge, practical communication, teamwork, leadership and critical thinking skills (Waters et al. 2012). In addition, the theoretical delivery of material without practical support impacts skills in implementing disaster nursing (Shannon 2015; Durmus et al. 2026). However, disaster simulations are rarely conducted, and most activities are directed directly to the field without any scenario‐based briefing, so many learning processes are not carried out optimally. In practice, the limitations of simulation tools hinder the understanding of lecturers and students (Hawsawi et al. 2025). Furthermore, the COVID‐19 pandemic is one of the barriers to learning reported by students undergoing direct simulation practice, which impacts the less‐than‐optimal practical competency obtained.

From the perspective of disaster volunteers, it was found that nurses' competencies in disaster management have not been standardized. Many nurses lack adequate competencies in handling disasters across the pre‐disaster, disaster and post‐disaster phases. This deficiency is further exacerbated by limited learning materials and the lack of equipment used in the field. These findings are consistent with previous studies reporting that disaster nursing competencies are often inconsistently defined and insufficiently integrated into nursing education, resulting in gaps in preparedness and response capacity (Farokhzadian et al. 2024; Ličen and Prosen 2025).

Several disaster volunteers also observed that educators often lack sufficient mental preparedness, even though the curriculum should have addressed this aspect. This aligns with existing literature highlighting that psychological readiness and resilience are frequently overlooked components in disaster nursing education (Mert and Koksal 2024; Yanık and Ediz 2024). Additionally, participants noted significant variation in learning approaches across campuses, with some emphasizing emergency care rather than comprehensive disaster management. Similar inconsistencies have been identified in other contexts, where disaster‐related content is fragmented and not systematically embedded within curricula (Brinjee, Al Thobaity, Almalki, et al. 2021; Rostami et al. 2023). To date, competency standards for disaster nurses have not been officially established. This situation presents a significant challenge in producing graduates who are truly prepared to respond to disasters. Therefore, concrete efforts are needed to develop a standardized curriculum, strengthen the capacity of educators, and design relevant competency standards.

From the student side, there is a proposal to develop a virtual learning method to support disaster nursing education. This is also supported by Disaster Nurses Association representatives, who suggest using area‐based disaster risk analysis through the tabletop exercise method. Virtual simulations, including scenario‐based learning, virtual reality (VR) and computer‐based disaster drills, allow students to engage in realistic, high‐risk situations such as mass casualty incidents, triage decision‐making and emergency coordination without requiring extensive physical resources (Alsaqer and Hussein 2025; Andreatta et al. 2010; Calik et al. 2022; Ingrassia et al. 2015). This approach is considered to help participants understand the affected areas, potential hazards and contingency scenarios more concretely. Several students also stated that the tabletop is very useful because it allows them to understand the roles and responsibilities in disaster situations, primarily by dividing zones (green, yellow, red) (Emaliyawati, Ibrahim, Trisyani, et al. 2025). They consider simulation the most effective way to improve disaster preparedness (Emaliyawati, Ibrahim, Trisyani, et al. 2025). Disaster simulation is an important element in learning because it allows students to gain practical experience and integrate the theory they have learned (Hawsawi et al. 2025; Cunalata and Castillo 2025; Calisanie et al. 2025).

The development of a disaster nursing curriculum is essential to prepare nurses who are competent in addressing all phases of disaster management comprehensively. In the pre‐disaster phase, the curriculum focuses on strengthening promotive and preventive competencies through risk analysis, preparedness and community‐based education. The learning process is designed using an experiential learning approach, implemented through case studies, early disaster scenario simulations and active student engagement in community activities (Kolb 1984; United Nations 2024). In the emergency response phase, the curriculum emphasizes the development of clinical skills and rapid, accurate decision‐making in crisis situations, facilitated through high‐fidelity simulation, interprofessional scenarios and mass casualty management exercises (Kolb 1984; United Nations 2024).

In the post‐disaster phase, which has previously been identified as an underdeveloped area, the curriculum is strengthened by incorporating physical and psychosocial rehabilitation, continuity of care and a family‐centred care approach. Learning at this stage emphasizes critical reflection and experiential learning to enhance understanding of recovery processes and long‐term resilience (International Council of Nurses 2019). Overall, the integration across disaster phases is designed not only as a linear process but also as cyclical and reflective, enabling students to construct knowledge continuously through experience, reflection and conceptualization (Kolb 1984). Furthermore, collaboration with healthcare institutions, professional associations and disaster management agencies enhances the relevance and practical applicability of the curriculum in real‐world settings.

### 4.2 Potential and Opportunities for Disaster Nursing Learning

In addition to the barriers faced, the results of the FGD also revealed various strategic opportunities in the development of disaster nursing education in Indonesia. These opportunities arise from the geographical context of Indonesia, which is prone to disasters and is a rich and real source of learning for students.

The frequent occurrence of disasters in Indonesia provides authentic field experience, exposing the gap between theory and practice (Adriani et al. 2024). The FGD results also revealed that the direct involvement of students in visiting disaster locations is one of the most impactful learning approaches. The direct involvement of lecturers, volunteers and students in disaster situations becomes reflective material in restructuring the curriculum to be more contextual and responsive (Emaliyawati, Ibrahim, Trisyani, and Songwathana 2025). This real‐world experience‐based learning effectively increases nursing students' readiness and confidence in facing crises, including decision‐making, teamwork and adaptation to stress (Emaliyawati, Ibrahim, Trisyani, and Songwathana 2025). Students can witness firsthand the gap between theory and practice and gain concrete experiences that can strengthen their understanding and skills (Emaliyawati, Ibrahim, Trisyani, and Songwathana 2025). This shows that disaster events can be authentic learning resources for continuous curriculum improvement.

On the other hand, the synergy between educational institutions, scientific associations and disaster management organizations presents significant opportunities to enrich curricula and bridge academic learning with field practice. This collaboration enables students to learn directly from practitioners, engage in interprofessional and cross‐disciplinary training and better understand the role of nursing within the broader disaster management system. Previous studies have highlighted that community‐based and interprofessional learning approaches enhance disaster preparedness and strengthen collaborative competencies among nursing students (Garner et al. 2025; Calaguas 2025; Andriyanto and Hidayati 2021; Hill and Díaz 2024). Additionally, student involvement in SAU, independent campus programmes such as disaster mitigation, and exposure to experts from various disciplines are important strategies for developing holistic competencies. Evidence also suggests that experiential and learner‐centred approaches, including direct engagement in disaster‐related activities, significantly improve students' knowledge, skills and readiness (Hung et al. 2021, 2020). These opportunities indicate that disaster nursing education in Indonesia has strong potential for advancement, particularly through the integration of field experience, local wisdom and active student participation in disaster‐related initiatives.

From the lecturer's perspective, another potential opportunity is the involvement of disaster management institutions in the learning process. In addition, emergency lecturers who go directly to disaster locations can identify gaps between theory and practice in the field, which are then used as a basis for improving the curriculum. Previous studies have stated that the involvement of regional disaster management agencies in providing materials related to emergency evacuation and early warning during disasters to children with disabilities, teachers and parents increases teachers' and parents' understanding of disaster mitigation (Zulfiana et al. 2024). In addition, other opportunities and potentials that can be utilized are the active involvement of lecturers in the scientific association of disaster nurses, especially in developing disaster nursing materials. This view aligns with the perspective of disaster volunteers, who emphasize the importance of collaboration between educational institutions and scientific associations of disaster nurses in the student learning process. This synergy not only strengthens the alignment of teaching materials through standardized sources but also makes it easier for scientific associations to recruit students as nursing assistants in the field when a disaster occurs (Tayebi et al. 2024; Chan et al. 2010).

From the perspective of lecturers, disaster volunteers and students, one of the strategic potentials and opportunities in disaster learning is the existence of SAU. These units serve as platforms for developing student interests and enhancing their competencies. One student stated that disaster‐related materials delivered through SAU were easier to understand and visualize within a practical context. In addition, the Independent Learning programme in disaster mitigation significantly contributes to improving students' ability to respond to disasters, as it includes hands‐on disaster training.

In line with this, the scientific association emphasized that the most effective disaster learning is gained through direct experience either from real disaster events occurring around students or through simulations that closely resemble field conditions. This view was supported by volunteers, who considered simulations of various disaster types to be a valuable way of providing students with realistic insights. Additionally, students highlighted other learning opportunities, such as exposure to interdisciplinary speakers addressing current disaster issues. Project‐based activities involving guest lecturers, including those from international institutions, significantly broadened students' perspectives and understanding of disaster dynamics.

### 4.3 Strengths and Limitations of the Study

This study has several strengths that support the validity and relevance of the findings. First, the qualitative approach through FGD allows for an in‐depth exploration of various stakeholders' perceptions, experiences and needs in disaster nursing education. The involvement of four participant groups of lecturers, students, disaster volunteers and disaster nursing scientific associations provides a holistic and multi‐perspective picture of the issues studied. Second, participants come from various provinces and institutions in Indonesia, reflecting the diversity of geographical, institutional and experiential backgrounds, which enriches the data and strengthens the transferability of the findings to similar contexts.

However, this study also has several limitations. First, using FGDas the only data collection method may limit the exploration of personal experiences in more depth, which may be more optimal if combined with individual interviews. Second, group discussions conducted online due to pandemic restrictions may affect the quality of interaction and depth of discussion, especially in terms of non‐verbal expression and emotional involvement.

## 5 Conclusion

This study identifies critical gaps in disaster nursing education in Indonesia, particularly the lack of standardized curricula, limited resources and insufficient lecturer preparedness. Despite these challenges, substantial opportunities exist through experiential learning from frequent disaster events, strengthened collaboration with professional and disaster management institutions, and active student engagement. Addressing these issues requires the development of a standardized national curriculum, the integration of practice‐based learning approaches, and targeted capacity building for educators. Importantly, these findings provide evidence‐based insights for the Association of Indonesian Nursing Education Institutions to formulate and implement national competency standards for disaster nursing education. Such policy‐driven efforts are essential to enhance the preparedness and competence of future nurses in responding effectively to disaster situations.

## Funding

This publication was supported by Universitas Padjadjaran through the Indonesian Endowment Fund for Education (LPDP), under the Ministry of Higher Education, Science, and Technology of the Republic of Indonesia, and administered through the EQUITY Program (Contract Nos. 4303/B3/DT.03.08/2025 and 3927/UN6.RKT/HK.07.00/2025).

## Ethics Statement

This research was conducted based on the principles of the Declaration of Helsinki (World Medical Association 2013). This study underwent a comprehensive ethical review process and received approval from the Ethics Committee of the Faculty of Medicine, Universitas Padjadjaran. The ethical registration number is as follows: 1180/UN6.KEP/EC/2019. All procedures in this research adhered to the ethical principles for research involving human participants. Prior to the interviews, all participants were informed about the study objectives, procedures, potential risks and their rights as participants. Written informed consent was obtained from each participant before the interviews began. Participants were also assured that they could withdraw from the study at any time without any consequences. The interviews were considered safe, as all participants were in stable condition and not experiencing illness. This study ensures the anonymity, confidentiality and security of all data collected. The authors confirm that all participants consented to the anonymous publication of their responses.

## Conflicts of Interest

The authors declare no conflicts of interest.

## Data Availability

The data that support the findings of this study are available on request from the corresponding author. The data are not publicly available due to privacy or ethical restrictions.
